# Innovative Approaches and Therapies to Enhance Neuroplasticity and Promote Recovery in Patients With Neurological Disorders: A Narrative Review

**DOI:** 10.7759/cureus.41914

**Published:** 2023-07-15

**Authors:** Jitesh Kumar, Tirath Patel, Fnu Sugandh, Jyotishna Dev, Umesh Kumar, Maham Adeeb, Meet Popatbhai Kachhadia, Piyush Puri, FNU Prachi, Mohammad Uzair Zaman, Satesh Kumar, Giustino Varrassi, Abdul Rehman Shah Syed

**Affiliations:** 1 Internal Medicine, Ghulam Muhammad Mahar Medical College, Sukkur, PAK; 2 Medical Student, American University of Antigua, St. John's, ATG; 3 Medicine, Ghulam Muhammad Mahar Medical College, Sukkur, PAK; 4 Medicine, Civil Hospital Karachi, Karachi, PAK; 5 Pediatric Medicine, Green City Hospital, Kathmandu, NPL; 6 Internal Medicine, TUTH (Tribhuvan University Teaching Hospital) Institute Of Medicine, Kathmandu, NPL; 7 Medicine and Surgery, Dow University of Health Sciences, Karachi, PAK; 8 Medicine, Dow University of Health Sciences, Karachi, PAK; 9 Internal Medicine, PDU (Pandit Deendayal Upadhyay) Medical College, Civil Hospital Campus, Rajkot, IND; 10 Internal Medicine, Adesh Institute of Medical Science and Research, Bathinda, IND; 11 Medicine, Guru Teg Bahadur Hospital, Delhi, IND; 12 Medicine, Bacha Khan Medical College, Mardan, PAK; 13 Medicine and Surgery, Shaheed Mohtarma Benazir Bhutto Medical College, Karachi, PAK; 14 Pain Medicine, Paolo Procacci Foundation, Rome, ITA

**Keywords:** traumatic, stroke, neurodegenerative, neurological, neuroplasticity, rehabilitation, brain

## Abstract

Brain rehabilitation and recovery for people with neurological disorders, such as stroke, traumatic brain injury (TBI), and neurodegenerative diseases, depend mainly on neuroplasticity, the brain's capacity to restructure and adapt. This literature review aims to look into cutting-edge methods and treatments that support neuroplasticity and recovery in these groups. A thorough search of electronic databases revealed a wide range of research and papers investigating several neuroplasticity-targeting methods, such as cognitive training, physical activity, non-invasive brain stimulation, and pharmaceutical interventions. The results indicate that these therapies can control neuroplasticity and improve motor, mental, and sensory function. In addition, cutting-edge approaches, such as virtual reality (VR) and brain-computer interfaces (BCIs), promise to increase neuroplasticity and foster rehabilitation. However, many issues and restrictions still need to be resolved, including the demand for individualized treatments and the absence of defined standards. In conclusion, this review emphasizes the significance of neuroplasticity in brain rehabilitation. It identifies novel strategies and treatments that promise to enhance recovery in patients with neurological illnesses. Future studies should concentrate on improving these therapies and developing evidence-based standards to direct clinical practice and enhance outcomes for this vulnerable population.

## Introduction and background

In the fields of neuroscience and rehabilitation, there has been a significant increase in interest regarding neuroplasticity, which refers to the brain's extraordinary ability to undergo changes and reorganization. It speaks to the ability of the brain to adapt its form and function in response to outside stimuli, knowledge, and experience. Neuroplasticity is crucial to brain rehabilitation to recover and regain function following neurological insults, such as stroke, traumatic brain injury (TBI), or neurodegenerative illnesses [1]. This in-depth narrative review explores cutting-edge methods and treatments that support neuroplasticity and encourage recovery in people with these neurological illnesses.

A key factor underlying brain adaptability and resilience is neuroplasticity. The brain's ability to alter was once thought to be restricted to crucial stages of development. However, recent studies have revealed that neuroplasticity lasts throughout a person's life [2]. This newfound knowledge has completely changed how brain rehabilitation is practiced by opening new doors for treatment and recovery. It is impossible to overestimate the significance of neuroplasticity in brain recovery. Following a brain injury or illness, it is the foundation for functional recovery and remodeling [2,3]. By rerouting neuronal pathways, creating new connections, and enlisting alternate regions to carry out disrupted duties, neuroplasticity enables the brain to make up for damaged areas. Individuals with neurological diseases may experience amazing healing if neuroplasticity is harnessed and enhanced through focused therapies.

Stroke is a neurological condition that can be treated using neuroplasticity-based methods. Blood flow to the brain is disrupted during a stroke, which causes damage to brain tissues, and is one of the leading causes of disability globally. Motor, linguistic, cognitive, and sensory processing deficits are common in stroke survivors [4]. By promoting healing and functional restoration, neuroplasticity is essential to stroke rehabilitation. Neuroplasticity can be tapped into through a variety of treatments, including virtual reality (VR), transcranial magnetic stimulation (TMS), constraint-induced movement therapy (CIMT), and pharmaceutical interventions. Another neurological illness for which neuroplasticity-based treatment strategies have shown promise is TBI. TBI happens when an outside force damages the brain, which results in cognitive, behavioral, and physical problems; the capacity of the brain to heal and remodel itself after TBI is supported by neuroplasticity [4,5]. To activate neuroplastic changes and advance rehabilitation in people with TBI, cognitive training programs and neuromodulation methods, including repetitive TMS (rTMS) and transcranial electrical stimulation (TES), and pharmaceutical therapies are used. These treatments focus on particular brain circuits, encourage synaptic plasticity, and improve cognitive abilities [5].

Neuroplasticity-based treatments are also advantageous for neurodegenerative disorders, such as Alzheimer's disease, Parkinson's disease, and multiple sclerosis. These conditions cause the progressive degradation of brain tissues, which worsens cognitive function, impairs movement, and causes other distressing symptoms [5]. Emerging research reveals that the brain retains some degree of plasticity even in pathology, even though neurodegenerative disorders are frequently characterized by neuroplasticity impairment. Modulating neuroplasticity, halting disease development, and easing symptoms have all been proven possible with pharmaceutical therapies, cognitive training, physical exercise, and non-invasive brain stimulation techniques (e.g., TMS and tDCS) [5,6].

Finally, neuroplasticity is a crucial component of brain rehabilitation in people with neurological conditions, such as stroke, TBI, and neurodegenerative illnesses. Understanding the mechanisms and principles of neuroplasticity lays the groundwork for creating novel strategies and treatments to speed healing and encourage functional recovery [6]. Neuroplasticity-based therapies give people with neurological illnesses hope and promise by taking advantage of the brain's extraordinary capacity to rearrange itself. The mechanics, effectiveness, and potential future directions in neuroplasticity and brain rehabilitation will all be examined in this narrative review, which will delve deeply into these cutting-edge methods and treatments [7].

## Review

Neuroplasticity mechanisms and principles (synaptic plasticity, structural plasticity, and principles of neuroplasticity)

Neuroplasticity is a key characteristic of the nervous system or the brain's capacity to reconfigure and adapt in response to external stimuli and experiences. It includes a wide range of systems and ideas that support the brain's ability to adapt, learn, and establish memories. Understanding the mechanisms and principles of neuroplasticity offers insightful knowledge of the dynamic nature of the brain and makes it possible to consider various therapeutic approaches for treating neurological illnesses and brain rehabilitation [8]. This concise and thorough overview addresses the fundamental concepts behind synaptic plasticity, structural plasticity, and the processes that underlie neuroplasticity. Synaptic plasticity is a key mechanism underlying neuroplasticity, which enables the brain to change the potency and effectiveness of synaptic connections between neurons. Two main types of synaptic plasticity, long-term potentiation (LTP) and long-term depression (LTD), are essential for learning and memory functions. LTP strengthens a synapse's connection, resulting in improved synaptic transmission [8,9]. It is thought that strengthening synapses allows information to be stored and memories to be created. Meanwhile, LTD is the deterioration of synaptic connections brought on by low-frequency stimulation or prolonged inactivity. Synaptic pruning and refinement are made possible by LTD, which shapes neuronal circuits to maximize their effectiveness and adapt to shifting environmental demands [9].

The physical alterations that take place in the neuronal architecture of the brain are included in structural plasticity. It involves alterations to the connectivity and architecture of neurons, including neurogenesis, dendritic remodeling, and axonal sprouting. Developing new branches and connections from preexisting axons is referred to as axonal sprouting. In reaction to injury or changes in functional requirements, this mechanism creates new brain circuits and information rerouting. The rewiring of neuronal networks is made possible by dendritic remodeling, which involves changes to the branching patterns and synaptic connections of dendrites [10]. The birth of new neurons, or neurogenesis, occurs in particular parts of the brain, including the hippocampus, and it helps people learn, remember things, and heal from brain injuries. The rules and mechanisms governing the adaptation of the brain can be understood using the principles of neuroplasticity. Hebbian plasticity is one such principle based on the idea that "neurons that fire together wire together." According to Hebb’s theory, synaptic connections become stronger when two neurons are frequently stimulated, simultaneously strengthening their connection. Associative learning and the growth of new brain pathways depend on Hebbian plasticity [10,11].

Another theory that emphasizes how activity and experience shape brain connections is use-dependent plasticity. It claims that although neuronal circuits that are infrequently active degrade, those that are frequently used or stimulated grow stronger and more effective [11]. This idea emphasizes the need for exposure and consistent learning and skill development practice. The plasticity of plasticity itself is referred to as metaplasticity. It includes modifying the brain's receptivity to additional variations in synaptic strength. Depending on previous activity levels and the history of synaptic modifications, metaplasticity processes can increase or decrease the ability for subsequent neuroplastic changes [11,12]. According to this theory, the last neural activity and plasticity can affect how sensitive the brain is to present and future inputs.

In conclusion, neuroplasticity refers to various mechanisms and principles that allow the brain to change its structure and make new connections in response to experiences and external stimuli. Learning and memory functions are shaped by synaptic plasticity, which also includes LTP and LTD. Structural plasticity makes the restructuring of brain networks and new connections possible, enabling axonal sprouting, dendritic remodeling, and neurogenesis [12]. Hebbian plasticity, use-dependent plasticity, and metaplasticity are a few examples of neuroplasticity concepts that shed light on the principles that control the brain's adaptability. It is crucial to comprehend these mechanisms and principles to create therapeutic strategies to support brain rehabilitation and treat neurological illnesses.

Neuroplasticity in stroke rehabilitation

Understanding Stroke-Induced Neural Damage and Potential for Recovery

The primary cause of long-term disability, stroke, is caused by a disruption in the blood supply to the brain, which causes functional limitations and neurological damage [13]. The location and intensity of the stroke affect the degree of brain damage. The brain has an innate neuroplasticity capacity, making potential recovery and functional restoration possible despite the initially devastating effects. Effective rehabilitation solutions must consider the mechanics of neurological injury and the brain's ability to heal.

CIMT

CIMT is a rehabilitation method that encourages motor recovery in stroke victims by utilizing the concepts of neuroplasticity [13,14]. To promote the use of the injured limb, CIMT entails restraint of the entire limb. This stimulates brain pathways and aids in the rebuilding of motor circuits. CIMT encourages functional gains, improves motor control, and develops neuroplastic changes in the brain by confining the unaffected limb and intensely exercising the affected limb [14].

TMS and Transcranial Direct Current Stimulation (tDCS)

In stroke rehabilitation, the non-invasive brain stimulation techniques TMS and transcranial direct current stimulation (tDCS) are utilized to modulate neuroplasticity [15]. Using magnetic pulses applied to the scalp, TMS can selectively activate or inhibit neuronal circuits in the brain by causing localized electrical currents. tDCS modifies the excitability of neurons by administering a weak electrical current through electrodes positioned on the scalp. Both methods can potentially improve neuroplasticity and speed motor recovery in stroke patients.

VR and Gamified Rehabilitation

The use of VR and gamified rehabilitation, which take advantage of the concepts of neuroplasticity to improve motor recovery and functional results, has emerged as novel methods to stroke rehabilitation [15,16]. VR offers a dynamic environment that can imitate real-world events and motivate and challenge patients. VR-based therapy fosters neuroplastic changes, stimulates active engagement, and improves motor learning and recovery by including motor activities, feedback, and rewards.

Pharmacological Interventions Targeting Neuroplasticity

The use of pharmaceutical therapies to promote neuroplasticity and speed stroke recovery has also been investigated [16]. Many medications can influence neuroplasticity processes, such as boosting synaptic transmission, facilitating synaptogenesis, or regulating neurotransmitter levels [17]. For instance, the ability of selective serotonin reuptake inhibitors (SSRIs) to improve neuroplasticity and functional recovery in stroke patients has been studied. Neurotrophic factors have also been investigated for their potential to support neuronal survival, plasticity, and active recovery. One such factor is brain-derived neurotrophic factor (BDNF).

In conclusion, neuroplasticity is essential to stroke recovery, providing chances for healing and functional advancements. Utilizing the concepts of neuroplasticity, methods, such as CIMT, TMS, and tDCS, improve motor recovery [17]. Gamified treatment and VR offer immersive environments encouraging neuroplastic changes and motor development. Pharmacological therapies that target neuroplasticity show promise in speeding up stroke recovery by regulating synaptic plasticity, enhancing neuronal survival, and boosting functional recovery. It is possible to significantly improve stroke rehabilitation outcomes and the quality of life for stroke victims by combining these methods with conventional therapy strategies [17,18].

Neuroplasticity in TBI rehabilitation

Pathophysiology and Challenges of TBI Recovery

The complicated neurological disorder known as TBI is characterized by brain damage brought on by an external force, such as a blow to the head or a penetrating injury [18]. Cellular damage, inflammation, oxidative stress, and excitotoxicity are some of the primary and secondary injury mechanisms involved in TBI pathophysiology. These mechanisms bring a variety of physical, cognitive, and behavioral deficits, which also contribute to the disruption of brain circuits. Due to the considerable neuronal damage and the complex structure of the brain's repair mechanisms, TBI rehabilitation can be complex [18,19].

Cognitive Training and Rehabilitation Strategies

People with TBI frequently experience cognitive deficits, which can profoundly affect everyday functioning and quality of life. Mental training and rehabilitation procedures are used to improve neuroplasticity and encourage recovery in cognitive domains, such as attention, memory, executive function, and problem-solving [19]. These methods entail systematic and specific exercises that excite and challenge neural networks and encourage adaptive changes in the brain. According to the demands and objectives of the individual, cognitive rehabilitation may use methods, including attentional control training, memory retraining, and problem-solving activities [20].

Neuromodulation Techniques: rTMS and TES

TES and rTMS are two neuromodulation techniques that have shown promise as treatments for TBI patients [21]. These approaches aim to increase neuroplasticity and speed up rehabilitation. To modulate neuronal activity and promote neuroplastic changes, rTMS entails delivering magnetic pulses to particular brain areas. In TBI rehabilitation, it has been investigated to focus on cognitive deficits, emotion control, and motor recovery. At the same time, TES modifies neuronal excitability and encourages neuroplasticity by delivering weak electrical currents through electrodes placed on the scalp. Both rTMS and TES have demonstrated promise for improving cognitive performance, motor recovery, and overall functional results in people with TBI [22].

Pharmacological Interventions to Enhance Neuroplasticity in TBI

Pharmacological approaches that affect neuroplasticity have been researched as potential complementary therapies to improve functional outcomes and recovery in people with TBI. Numerous medications have demonstrated the potential to influence neuroplasticity processes, such as increasing brain repair, promoting synaptic plasticity, and lowering inflammation [22]. For instance, the potential to support neuronal survival, synaptic plasticity, and cognitive recovery has been explored concerning specific neurotrophic factors, such as BDNF. Several pharmaceutical substances, such as memantine and amantadine, have been investigated in TBI rehabilitation for their neuroprotective and cognitive-improving benefits. Additional study is required to ascertain the ideal timing, dosage, and tailored strategies for pharmacological therapies in TBI [22,23].

In conclusion, neuroplasticity is essential for TBI rehabilitation because it provides chances for healing and functional advancements, as well as strategies for cognitive training and rehabilitation attempt to improve cognitive deficits by encouraging neural network adaptation [23]. In people with TBI, neuromodulation methods, including TES and rTMS, modify brain activity and improve neuroplasticity. Through mechanisms, such as synaptic plasticity, neuroprotection, and decreased inflammation, pharmacological therapies show promise in enhancing neuroplasticity and fostering recovery. These methods could be combined with interdisciplinary rehabilitation programs to improve TBI rehabilitation outcomes and the quality of life for those who suffer from this severe neurological illness [24].

Neuroplasticity in neurodegenerative diseases

In particular brain regions, progressive neuronal loss and degeneration are hallmarks of neurodegenerative illnesses, such as Alzheimer's, Parkinson's, and Huntington's diseases. Due to the cognitive and motor impairments they cause and the general loss of quality of life, these diseases provide substantial obstacles. However, recent studies have shown that neuroplasticity may play a part in reducing the consequences of neurodegeneration and encouraging functional recovery [25]. The processes of neurodegeneration, the impairment of neuroplasticity, and numerous therapies targeted at utilizing neuroplasticity in neurodegenerative illnesses are all covered in this concise yet thorough overview.

Mechanisms of Neurodegeneration and Neuroplasticity Impairment

Complex mechanisms are involved in neurodegenerative illnesses, contributing to the progressive loss of synaptic connections and neurons. Among these mechanisms include the buildup of aberrant protein aggregates, oxidative stress, neuroinflammation, mitochondrial dysfunction, and compromised cellular clearance systems. As neurodegeneration worsens, it can reduce neurotrophic support, disrupt synaptic plasticity, and change the overall structural integrity of brain circuits, all of which can affect neuroplasticity [25,26]. Neurodegenerative disorders' symptoms and courses are made worse by the impairment of neuroplasticity.

Physical Exercise and Aerobic Training

Exercise has become recognized as a technique for enhancing neuroplasticity and delaying the onset of neurodegenerative illnesses. In particular, aerobic exercise has been found to promote brain health, increase neuroplasticity, and slow cognitive decline. Regular practice encourages the release of growth factors, including BDNF, which supports neuronal survival, synaptic plasticity, and the production of new neurons. Along with sustaining cognitive function and lowering the risk of neurodegenerative disorders, exercise improves cerebral blood flow and boosts neurogenesis and general brain connectivity [27].

Cognitive Training and Brain Fitness Programs

Mental training and brain fitness programs are used to improve cognitive function and increase neuroplasticity in people with neurodegenerative disorders. Memory, attention, problem-solving, and language skills are just a few of the cognitive functions tested in these programs' structured and focused tasks. People can encourage neuroplastic changes, fortify neural networks, and possibly compensate for the cognitive deficits brought on by neurodegenerative disorders by engaging in intellectually challenging activities [28]. It has been demonstrated in numerous studies that cognitive training programs can enhance cognitive performance, functional independence, and quality of life in people with Alzheimer's disease and other neurodegenerative conditions.

Non-Invasive Brain Stimulation Techniques in Neurodegenerative Diseases

TMS and tDCS, two non-invasive brain stimulation methods, are potential therapies to modify neuroplasticity and ameliorate symptoms in neurodegenerative illnesses [27,28]. TMS alters neuronal activity and fosters neuroplastic changes by applying magnetic pulses to particular brain areas. Meanwhile, tDCS modifies the excitability of neurons by delivering a weak electrical current through electrodes positioned on the head. In people with neurodegenerative disorders, these methods can potentially enhance motor symptoms, cognitive function, and overall quality of life.

Potential Neuroprotective Effects of Pharmacological Agents on Neuroplasticity

The potential neuroprotective effects of pharmaceutical therapies targeting neuroplasticity in neurodegenerative illnesses have been studied. These drugs promote neurotrophic support, lessen inflammation, increase synaptic plasticity, and enhance general brain health [28,29]. For instance, it has been demonstrated that medications that affect the cholinergic system, such as acetylcholinesterase inhibitors, improve cognitive function in patients with Alzheimer's disease. As possible treatments to boost neuroplasticity and slow neurodegeneration, neurotrophic factors, such as BDNF, and other substances that support neuronal survival and synaptic plasticity are also being investigated.

Finally, neuroplasticity shows promise in reducing the consequences of neurodegenerative illnesses and encouraging functional recovery. Programs for brain fitness, cognitive training, and physical activity provide non-pharmacological ways to improve neuroplasticity and maybe slow the onset of dementia [30]. Techniques for non-invasive brain stimulation, such as TMS and tDCS, offer ways to control neuroplasticity and alleviate symptoms of neurodegenerative disorders. Drugs that target the processes involved in neuroplasticity may also have neuroprotective effects and aid in functional recovery. Researchers and doctors can create creative approaches to improve the lives of people with neurodegenerative disorders and potentially delay the advancement of the disease by comprehending and utilizing the principles of neuroplasticity [28-30].

Innovative technologies and neurorehabilitation

By using a variety of treatment methods, neurorehabilitation seeks to facilitate healing and functional gains in people with neurological diseases. New pathways for boosting neuroplasticity and enhancing rehabilitation outcomes have been made possible by novel technologies in recent years, revolutionizing the area of neurorehabilitation [31]. This succinct and thorough overview explores some of the cutting-edge technologies used in neurorehabilitation, such as brain-computer interfaces (BCIs) and neurofeedback, robotic-assisted therapy, and exoskeletons, VR and augmented reality (AR) applications, wearable technology, and sensor-based technologies [31].

BCIs and Neurofeedback

Bypassing conventional cerebral channels, BCIs allow direct communication between the brain and an external device. By allowing users to manipulate external objects or interfaces using their brain activity, BCIs can be utilized to improve neurorehabilitation [31,32]. For people with severe motor disabilities, this technology has much promise because it will give them back control over their surroundings. A type of BCI known as neurofeedback teaches users how to self-regulate their brain patterns by giving them immediate feedback on their brain activity. By encouraging self-regulation of brain function and improving neuroplasticity, neurofeedback has demonstrated promise in treating several neurological diseases, including stroke, TBI, and attention deficit hyperactivity disorder (ADHD) [32].

Robotic-Assisted Therapy and Exoskeletons

Robotic equipment is used in robotic-assisted treatments to facilitate movement and rehabilitation exercises. Individuals can conduct repetitive and focused actions owing to these gadgets' exact and customizable support or resistance. Individuals with motor deficits brought on by a stroke, spinal cord injury, or other neurological diseases may benefit significantly from robotic-assisted therapy [33]. Wearable external structures called exoskeletons are a form of robotic technology that support and improve the body's movement. Exoskeletons can aid people with mobility disabilities to restore their walking abilities and increase their overall functional independence by offering assistance or resistance during gait training [33,34].

VR and AR Applications in Neurorehabilitation

Due to its immersive and interactive features, VR and AR technologies have attracted much attention in neurorehabilitation [35]. AR places digital information over the real world, whereas VR creates a virtual environment. For therapeutic exercises and activities, these technologies offer surroundings that are interesting and inspiring. VR and AR have a variety of applications in neurorehabilitation, including the improvement of motor function, the development of cognitive abilities, and the stimulation of the senses. People with cognitive impairments can participate in mental training exercises using AR interfaces. By contrast, stroke patients can use VR systems to practice reaching and grasping actions in a virtual environment [36].

Wearable Devices and Sensor-Based Technologies for Monitoring and Enhancing Neuroplasticity

The capacity to continuously monitor and track different aspects of neurorehabilitation is made possible by wearable technology and sensor-based technologies, which offer physicians and patients helpful input. With the help of these technologies, tailored rehabilitation therapies are potential. Data on movement patterns, physiological characteristics, and daily activities can be captured [35,36]. Smart watches, fitness trackers, and motion sensors are examples of wearable technology that can provide real-time feedback, promote engagement, and track development during therapeutic exercises. Electromyography (EMG) sensors and electroencephalography (EEG) headsets are examples of sensor-based technologies that can accurately monitor muscle activity or brain signals, permitting focused therapies and neurofeedback [36].

In conclusion, cutting-edge technologies are revolutionizing the field of neurorehabilitation by offering fresh and efficient ways to promote neuroplasticity and boost rehabilitation results. Neurofeedback and BCIs provide direct brain-to-external-device communication. At the same time, robotic therapy and exoskeletons facilitate movement and rehabilitation exercises [37]. VR and AR applications produce interactive, immersive environments for therapeutic interventions. Continuous monitoring and individualized feedback are provided through wearable technology and sensor-based technologies. Clinicians and researchers can optimize treatment techniques, boost neuroplasticity, and ultimately enhance the quality of life for people with neurological diseases by incorporating this cutting-edge technology into neurorehabilitation programs [37,38].

Challenges and future directions in neuroplasticity research and practice

In neurological diseases, neuroplasticity, the brain's capacity to remodel and adapt in response to experiences and injuries, holds considerable promise for improving recovery and rehabilitation. At the same time, much has been learned about how to use neuroplasticity. However, several issues must be resolved before this subject can proceed [38]. This succinct and thorough overview examines some of the major issues and future directions in neuroplasticity research and practice, such as patient variability and customized treatment plans, the long-term sustainability of neuroplasticity gains, combining multiple modalities for synergistic effects, translating research findings into clinical practice, and ethical considerations and patient acceptance [39].

Patient Variability and Individualized Approaches

The wide range of individual responses to interventions and the intensity of neuroplastic alterations are two of the main difficulties in neuroplasticity research and practice. Everybody has a different brain, which is influenced by genetics, age, the type of injury, and environmental circumstances [40]. Therefore, it is essential to create tailored strategies that consider these elements to maximize the efficacy of therapies based on neuroplasticity. Neuroplasticity therapies can be more effective overall when customized to a person's unique requirements and features to maximize results [39,40].

Long-Term Sustainability of Neuroplasticity Gains

A challenge in neuroplasticity research is to ensure the long-term sustainability of neuroplastic alterations and the benefits made via rehabilitation. Despite the observation of short-term benefits, the ability to sustain and translate these advances into real-world functioning over the long term remains a substantial challenge [39,40]. Research is still being done to understand the mechanisms underlying long-term neuroplastic alterations better and to figure out how to encourage the consolidation and generalization of learned skills. To achieve long-lasting functional benefits, interventions, and support systems must be created that make it easier for neuroplastic alterations to be incorporated into daily activities.

Combining Multiple Modalities for Synergistic Effects

Researchers and clinicians are investigating the possible synergistic effects of combining several interventions and modalities to maximize neuroplasticity outcomes [23,24]. To simultaneously target several components of neuroplasticity, this strategy entails merging numerous treatments, such as physical therapy, cognitive training, neurostimulation, and pharmaceutical interventions. Through complementary mechanisms, combining modalities can accelerate neuroplastic changes and produce more significant and all-encompassing results in rehabilitation. However, figuring out the best mix and order of therapies for various neurological disorders and people still poses a challenge that necessitates more research [25-30].

Translation of Research Findings Into Clinical Practice

To make sure that the advantages of neuroplasticity research reach the people who can benefit from them, it is crucial to translate research findings into clinical practice. Although laboratory studies have produced encouraging results, several obstacles must be overcome before these therapies can be successfully used in clinical settings. Issues, including scarce resources, variations in therapeutic protocols, and the requirement for specific training and tools, can hamper the widespread use of neuroplasticity-based treatments [31-33]. Researchers, clinicians, policymakers, and funding organizations must work together to establish standardized protocols, disseminate knowledge, and make incorporating neuroplasticity interventions into standard clinical care easier to close the gap between research and practice.

Ethical Considerations and Patient Acceptance

The ethical issues surrounding their use and patient acceptance become more crucial as neuroplasticity-based therapies develop. The privacy and security of patient data gathered by neurotechnologies, potential hazards and adverse effects of treatments, and equal access to these technologies are all issues that need to be addressed by researchers and clinicians [23,26,28]. In addition, the success of neuroplasticity-based methods is greatly influenced by the understanding and acceptance of patients. Fostering trust, assuring informed decision-making, and encouraging active engagement in rehabilitation all depend on educating patients and caregivers about the underlying science, potential advantages, and reasonable expectations of neuroplasticity therapies.

Although neuroplasticity presents intriguing opportunities for enhancing recovery and rehabilitation in people with neurological diseases, several issues must be resolved to realize its potential fully. Key topics that demand ongoing research, collaboration, and innovation include individualized treatments, long-term sustainability, modality combinations, translation into clinical practice, and ethical considerations [22-25]. Researchers and clinicians must address these issues to fully utilize neuroplasticity and open the door to more efficient and individualized interventions that enhance the lives of people with neurological diseases.

Key findings and developments in neuroplasticity-based brain rehabilitation

The extraordinary capacity of the brain to adapt and rearrange itself, known as neuroplasticity, has been identified as a possible pathway for brain rehabilitation in people with neurological diseases. Understanding the mechanisms underpinning neuroplasticity and utilizing its potential to aid recovery and functional improvements have come a long way. This conclusion includes a summary of the most important discoveries and developments in neuroplasticity-based brain rehabilitation and a list of viable strategies and possible future directions in this fascinating area.

Summary of Important Findings

Synaptic plasticity (e.g., LTP and LTD) and structural plasticity (e.g., axonal sprouting, dendritic remodeling, and neurogenesis) have been studied as neuroplasticity mechanisms. These pathways are essential for creating new connections, rewiring brain circuits, and fostering functional recovery.

Patient Variability

It is critical to acknowledge that every person reacts to interventions based on neuroplasticity differently. Elements, such as heredity, age, the type of damage, and environmental factors, can influence the degree and rate of neuroplastic alterations. Optimizing rehabilitation outcomes requires adjusting approaches to individual needs and features.

Innovative Technologies

Neuroplasticity-based rehabilitation has been transformed by innovative technologies, such as BCIs, robotic treatment, VR, and wearable gadgets. BCIs provide direct brain-to-external-device communication, whereas robotic therapy offers precise resistance or help during workouts. Wearable technology enables continuous monitoring and individualized feedback, while VR delivers immersive environments for therapeutic interventions. Combining different modalities has the potential to improve neuroplasticity outcomes. Such modalities include physical therapy, cognitive training, neurostimulation, and pharmaceutical therapies. These complementary strategies simultaneously address many facets of neuroplasticity, producing more detailed rehabilitative results.

Translation into Clinical Practice

A crucial step in maximizing the effectiveness of therapies based on neuroplasticity is the translation of research findings into customary clinical practice. Standardized protocols, clinician and researcher collaboration, and knowledge dissemination are essential to close the knowledge gap between research and practice.

Summary of effective methods

Individualized Rehabilitation

The key to maximizing rehabilitation results is recognizing patient variability and adapting interventions to individual requirements and characteristics. Neuroplasticity-based interventions can be made as effective as possible by using personalized rehabilitation plans considering genetics, the type of injury, and environmental effects. Combination therapies promise to improve neuroplasticity outcomes by combining different modalities, such as physical therapy, cognitive training, and neurostimulation. Results in rehabilitation that are more thorough and effective can be achieved through synergistic effects attained through complementary methods.

Innovative Technology

Cutting-edge technology, including BCIs, robotic therapy, VR, and wearables, offer fresh ways to encourage neuroplasticity and improve the results of rehabilitation. These technological advancements provide immersive and engrossing experiences, exact monitoring and feedback, and the ability for focused treatments.

Translation and Implementation

For research findings to be implemented into ordinary clinical practice, it is essential to establish standardized methods, disseminate information, and promote collaboration between researchers, clinicians, policymakers, and funding organizations. This ensures that those who can benefit from neuroplasticity-based interventions receive those benefits.

Potential Courses of Action

Advanced neuroimaging methods: New neuroimaging methods, such as EEG, diffusion tensor imaging (DTI), and functional magnetic resonance imaging (fMRI), provide opportunities to study structural and functional changes in neuroplasticity better. These methods can help locate neuroplasticity biomarkers and customize therapies based on unique brain profiles. TMS and tDCS are examples of individualized brain stimulation techniques that can potentially improve neuroplasticity outcomes. These interventions can be more successful by adjusting the stimulation parameters per individual brain features.

Integrative rehabilitation models: Including mindfulness-based therapies, music therapy, and art therapy alongside neuroplasticity-based rehabilitation strategies may help people with neurological problems recover more completely. Investigating the interactions between these methods could result in brand-new, all-encompassing rehabilitation treatments.

Long-run sustainability: It is critical to address the issue of keeping neuroplasticity gains sustainable over the long run. The creation of support systems that make it easier for neuroplastic alterations to be incorporated into daily activities and continued study into the mechanisms that enable the consolidation and generalization of learned skills can result in long-lasting functional gains.

## Conclusions

There has been tremendous progress in the knowledge of the mechanisms, investigation of novel technologies, and individualization of interventions in neuroplasticity-based brain rehabilitation. Integrating modalities provides an optimistic view of the discipline, their application in clinical settings, and the investigation of potential future possibilities. The potential of neuroplasticity will be further unlocked by ongoing study, cooperation, and the incorporation of novel treatments, improving outcomes and quality of life for people with neurological diseases.
